# Generic Liquid Membranes for Electromembrane Extraction of Bases with Low or Moderate Hydrophilicity

**DOI:** 10.1021/acs.analchem.3c01052

**Published:** 2023-06-01

**Authors:** Chen Zhou, Samira Dowlatshah, Anne Oldeide Hay, Maria Schüller, Stig Pedersen-Bjergaard, Frederik André Hansen

**Affiliations:** † Department of Pharmacy, University of Oslo, P.O. Box 1068, Blindern, 0316 Oslo, Norway; ‡ West China School of Public Health and West China Fourth Hospital, Sichuan University, Chengdu 610041, China; § Department of Pharmacy, Faculty of Health and Medical Sciences, University of Copenhagen, Universitetsparken 2, 2100 Copenhagen, Denmark

## Abstract

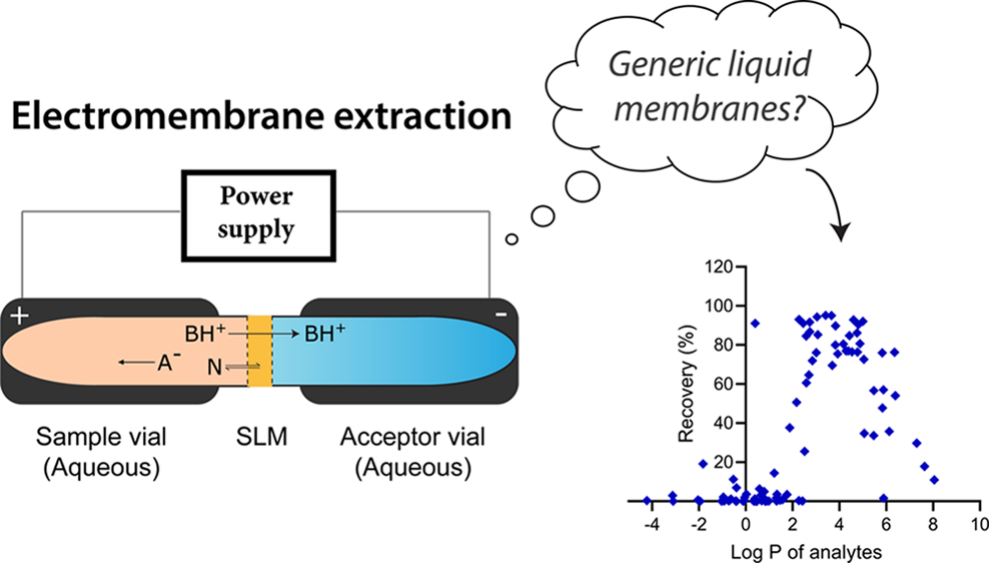

For the first time, this paper introduces the idea of generic extraction conditions in electromembrane extraction (EME), where the selection of the liquid membrane is based on the charge (*z*) and hydrophobicity (log *P*) of the analyte. A broad range of organic solvents were tested as liquid membranes, and 90 basic pharmaceuticals were used as model analytes (−4.2 < log *P* < 8.1). 2-Nitrophenyl octyl ether (NPOE) was confirmed as a highly efficient liquid membrane for mono- and dibases (+1.0 ≤ *z* ≤ +2.0) of low polarity in the log *P* range of 2.2–6.4. This log *P* range was set as the extraction window (operational range) of NPOE. NPOE provided very high operational stability. At 50 V, the current was at a 1 μA level, and gas formation and drifting pH due to electrolysis were insignificant. 2-Undecanone was discovered as a new and robust alternative. This solvent extracted monobasic analytes (*z* = +1) in the log *P* range of 1.0–5.8 and was efficient even for bases of moderate polarity. The current was at the 1–3 μA level when 2-undecanone was operated at 50 V. Tri(pentyl) phosphate emerged as another new alternative for bases in the log *P* range of 0.5 to 5.5, providing greater selectivity differences. This solvent provided a higher current (30–50 μA), but the EME system stability was not compromised. 2-Undecanone and tri(pentyl) phosphate extracted protonated bases mainly by hydrogen bond interactions. NPOE, on the other hand, extracted based on a combination of hydrogen bond and π-type interactions and was consequently less selective.

## Introduction

The development of analytical microextraction techniques has been a research-intensive area since the introduction of solid-phase microextraction (SPME) in 1990.^1^ As a result of this, a variety of microextraction techniques, in addition to SPME, are available and well-documented today, including (but not limited to) stir-bar sorptive extraction,^2^ microextraction in a packed syringe,^3^ single-drop microextraction,^4,5^ dispersive liquid–liquid microextraction,^6^ hollow-fiber liquid-phase microextraction,^7^ and electromembrane extraction.^8^ All these techniques provide very low consumption of chemical reagents, solvents, and materials and are therefore ideal for green sample preparation. In addition, due to the small dimensions, several of the microextraction techniques can be implemented in microchip systems.

In electromembrane extraction (EME), mass transfer is controlled by an electrical field; target analytes are extracted as charged species from the sample solution, across a liquid membrane, and into an aqueous acceptor. The electrical field is sustained across the liquid membrane and is provided by an external power supply. The liquid membrane is a 1–10 μL volume of an organic solvent (membrane solvent) immobilized in the pores of a porous polymeric membrane. Due to the small volume, the consumption of the organic solvent is reduced to a minimum, and this is a great advantage in terms of sustainability and greenness. Furthermore, acceptors are aqueous and can be analyzed directly by LC–MS and related techniques, without any evaporation and reconstitution steps. Finally, selectivity is unique in EME, as it is controlled by multiple parameters including the direction and magnitude of the electrical field, the chemical composition of the liquid membrane, and the pH.

Due to the unique properties of EME, a significant number (>400) of research papers have been published on this technique, reporting the extraction of pharmaceuticals,^9^ environmental contaminants,^10^ trace metals,^11^ and contaminants in food and beverages.^12^ Furthermore, there has been significant activity in the development of EME in different technical formats^13^ and in understanding the fundamentals and the principles of mass transfer.^14^ However, all this research has been conducted in a number of different laboratory-built systems, using a variety of experimental conditions. EME has the potential to establish as a general technique for microextraction of acids and bases, but to reach this level, commercial equipment and generic methods are required. Recently, commercial equipment for EME was launched, based on vials for samples and acceptors produced in a conducting polymer.^15^ However, generic methods are still missing.

For this reason, major research activities are currently in progress in the authors’ laboratory to develop a system of generic methods. Conceptually, this system will comprise a limited number of recommended extraction conditions, where the appropriate condition is selected based on the charge (*z*) and polarity (log *P*) of the analyte. Thus, based on *z* and log *P* as molecular descriptors, the system is intended to serve as a road map for EME. The work on this involves systematic studies of the extraction of large collections of basic and acidic model analytes, with a variety of organic solvents as the liquid membrane and under different conditions for the sample pH, acceptor, voltage, agitation, and extraction time. Based on all this information, which is obtained with commercial EME equipment, the experiences can be generalized for the first time.

Development of generic systems primarily concerns the selection of appropriate liquid membranes. The liquid membrane should (1) provide efficient extraction of compounds within a defined *z* and log *P* range (termed extraction window), (2) be stable during extraction, (3) and provide low current during operation. Criterion (1) is related to the solvation of analytes as ionic species in the liquid membrane, and this is a fine balance between the hydrophobicity of the liquid membrane and its molecular interactions with the analyte. Criterion (2) is related to the water solubility of the liquid membrane. Solvents with a solubility of >0.5 mg/mL tend to leak into the sample and acceptor during extraction, and this reduces the system stability. Water may also leak into the liquid membrane and further destabilize the system. The limit of 0.5 mg/mL is equivalent to the solubility of 1-octanol, which through years of experience has been found to be one of the most water-soluble EME solvents that can yield stable systems. Criterion (3) is related to electrolysis, which occurs both in the sample and acceptor. Electrolysis is not a practical issue at low currents (<50 μA), but at higher currents, drifting pH and bubble formation in the sample and acceptor may affect the extraction performance. 50 μA is selected as the recommended limit as this historically is well within a margin of safety.^16^ All criteria should be fulfilled for both neat standard samples and complex matrix samples.

The current paper is the first one reporting on the development of generic extraction conditions for EME. The work was focused on bases of low and moderate polarity (log *P* > 2 and 0 ≤ log *P* ≤ 2, respectively). 2-Nitrophenyl octyl ether (NPOE) has more or less been established as the primary liquid membrane in EME,^14,17^ but a variety of experimental conditions have been used in the literature, and data are therefore not comparable. NPOE was tested herein for the simultaneous extraction of 90 basic model analytes from aqueous buffer and human plasma samples, and from this large set of experimental data, the extraction window was established in terms of *z* and log *P*. NPOE is not efficient for bases of moderate polarity; therefore, a large number of alternative solvents were tested. Among these, 2-undecanone was found to be superior, with tri(pentyl) phosphate as an alternative. Liquid membranes were recommended by evaluation of the before mentioned criteria. The current study is limited to characterizing generic liquid membranes. Generic conditions of other experimental parameters will be presented in forthcoming research.

## Experimental Section

### Chemicals and Reagents

Hexadecane, pentyl benzene, 2-decanone, 2-undecanone, 6-undecanone, tris(2-butoxyethyl) phosphate, tri(butyl) phosphate, tri(pentyl) phosphate, tris(2-ethylhexyl) phosphate, bis(2-ethylhexyl) phosphite, benzyl 2-nitrophenyl ether, 2-nitrophenyl pentyl ether, dihexyl ether, 2-nitrophenyl octyl ether (NPOE), 2-ethyl nitrobenzene, iodopentafluoro benzene, 2-nitro-cumene, dodecyl acetate, dimethyl sulfoxide (DMSO), acetonitrile (LC–MS grade), and methanol (LC–MS grade) were obtained from Sigma-Aldrich (St. Louis, MO, USA). Formic acid (LC–MS grade) was purchased from VWR (Radnor, PA, USA). Circular polypropylene membranes (type PP2E) with a thickness of 110 μm and a diameter of 9 mm were from Extraction Technologies Norway (Ski, Norway).

In total, 90 different drugs and endogenous metabolites, with log *P* ranging from −4.2 to 8.1, were used as model analytes (Table S1, Supporting Information). The model analytes were dissolved individually in methanol, DMSO, or water, and the corresponding stock solutions were from 1.0 to 9.0 mg/mL. From these, a mixture of all 90 compounds at 5 μg/mL was prepared and split into aliquots and stored at −28 °C. Working solutions used for extraction experiments were prepared by spiking to 100 ng/mL.

Extraction experiments were conducted with either spiked 100 mM formic acid or human plasma. Human plasma was obtained from Oslo University Hospital (Oslo, Norway) and stored at −28 °C. Before extraction, plasma was diluted 1:1 (v/v) with 200 mM formic acid. Ultrapure water used in the experiments was obtained with a Millipak (0.22 μm filter) Milli-Q water purification system (Molsheim, France). All chemicals and reagents were of analytical reagent grade unless otherwise noted.

### EME Equipment

In this study, a prototype device for conductive vial electromembrane extraction from Extraction Technologies Norway (Ski, Norway) was used to perform the EME experiments.^15^ Photos of the equipment are provided in Figure S1. In this technical format of EME (conducting vial EME), vials were used to hold sample and acceptor solutions in a working volume of 200–500 μL. The total inner vial volume was 600 μL. The vials were produced in a conducting polymer, and in this way, they also served as electrodes. A circular porous polypropylene membrane (Extraction Technologies Norway) was used as the support membrane, and this was placed in a tailor-made support membrane union. The latter served to assemble the extraction cell, where the sample vial, the liquid membrane, and the acceptor vial were connected. Up to 10 extraction cells were loaded into a 10-position holder (Extraction Technologies Norway), where the conducting vials came in contact with an electrode to provide the electrical field. The 10-position holder was mounted on a shaking board for sample agitation during extraction. Voltage was supplied by a model ES 0300-0.45 power supply from Delta Elektronika BV (Zierkizee, Netherlands). A Fluke 287 multimeter (Everett, WA, USA) was used for current monitoring during extraction.

### EME Procedure

For each extraction cell, 250 μL of sample and 250 μL of acceptor solution (100 mM formic acid) was filled into the sample and acceptor vial, respectively. The sample comprised neat solution (100 mM formic acid) or plasma diluted 1:1 with 200 mM formic acid, with analytes spiked into it at 100 ng/mL. No protein precipitation was observed during acidification of plasma. Then, the polypropylene support membrane was placed in the support membrane union and fixed by screwing in the acceptor vial. A 10 μL volume of the liquid membrane solvent was deposited onto the support membrane and became immobilized in the pores. Subsequently, the sample vial was connected to complete the assembly of the entire extraction cell. The extraction cell was placed in the 10-position holder of the EME device such that the sample vial was connected to the positive electrode (anode) while the acceptor vial was connected to the negative electrode (cathode). Extraction was performed by application of 50 V for 30 min and with 750 rpm horizontal agitation. The agitation rate was selected by previous characterization of its relationship with sample/acceptor volumes.^18^ After the extraction, the acceptor solution was collected and analyzed by LC–MS/MS.

### LC–MS/MS Analysis

LC–MS/MS analysis was performed with an Agilent 1290 Infinity II UHPLC system (Agilent Technologies, Santa Clara, CA, USA), consisting of a binary pump, an autosampler, and a column compartment with controllable temperature. An Eclipse Plus C18 column (2.1 mm × 50 mm, 1.8 μm, Agilent Technologies) maintained at 40 °C was used for separation. The injection volume was 1.0 μL. Mobile phases A and B comprised ultrapure water and acetonitrile in ratios of 5:95 and 95:5 (v/v), respectively, each with 0.1% formic acid. The following elution gradient was applied: mobile phase B was kept at 0% from 0.00 to 1.00 min, ramped to 53% from 1.00 to 6.00 min, ramped to 75% from 6.00 to 7.00 min and 100% from 7.01 to 7.50 min and was then set to 0% for a final 1.50 min re-equilibration. The flow of mobile phases was 0.4 mL/min during 0.00–7.00 min, 0.7 mL/min during 7.00–8.50 min, and 0.4 mL/min during 8.50–9.00 min.

Mass spectrometric detection was performed with an Agilent 6495 LC/TQ (Agilent Technologies) with positive electrospray ionization at 3 kV and with a gas temperature of 200 °C. The system was operated in the dynamic MRM mode, with a cycle time of 300 ms, resulting in a minimum dwell time of 4.52 ms. Further details about detection parameters are given in the Supporting Information (Table S1).

### Determination of Kamlet–Taft Solvent Parameters

For selected solvents, the Kamlet–Taft solvatochromic parameters, α (hydrogen bond acidity), β (hydrogen bond basicity), and π* (dipolarity/polarizability), were determined according to a previously reported method.^19^ For this, a UV–vis spectrophotometer (Evolution 201, Thermo Scientific, MA, US) scanning from 300 to 700 nm at 0.5 nm intervals was used.

### Computation of Physicochemical Properties

All reported physicochemical properties, such as log *P*, charge, and solubility, were calculated by Chemicalize (https://chemicalize.com, ChemAxon, Budapest, Hungary).

### Definitions and Calculations

Recovery (*R*) was calculated according to the following equation:
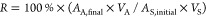
*A*_A,final_ is the peak area of the analyte in the acceptor after extraction, *V*_A_ is the volume of the acceptor, *A*_S,initial_ is the peak area of the analyte in the sample before extraction, and *V*_S_ is the volume of the sample. For extractions of the plasma sample, *A*_S,initial_ was replaced by *A*_postspiked matrix_, which is the peak area of the analyte in a postextraction spiked acceptor solution, after extraction of a blank matrix sample. Thus, the calculated recovery was independent of any matrix effects, namely, ion suppression or enhancement.^20^

The matrix effect (ME) was calculated according to the following equation.
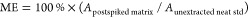
*A*_unextracted neat std_ is the analyte peak area of a neat standard of equal concentration to postspiked acceptor solution.

The extraction window for a given liquid membrane expresses the analyte log *P* range (from log *P*_Low_ to log *P*_High_) where high recoveries can be expected. Extraction windows were established based on experimental recovery data for 90 basic model analytes as follows:

Log *P*_Low_ is the lowest log *P* value where at least 50% of the model analytes in the range of log *P*_Low_ to log *P*_Low_ + 0.5 were extracted with recoveries above 40%.

Log *P*_High_ is the highest log *P* value where at least 50% of the model analytes in the range of log *P*_High_ – 0.5 to log *P*_High_ were extracted with recoveries above 40%.

The 40% criterion was selected based on experience over a long time in our laboratory, where recoveries above this limit normally give an RSD of <15%. Recoveries of ≥85% were defined as exhaustive.

## Results and Discussion

### Selection of the Analyte Charge (*z*) and Hydrophobicity (Log *P*) as Molecular Descriptors

The selection of the liquid membrane is a critical step during EME method development.^17^ For this reason, method development normally starts with testing different liquid membranes. Throughout the literature, it has been found that the selectivity of the liquid membrane, to a large extent, is related to the charge (*z*) and polarity (log *P*) of the analyte.^21,22^ Therefore, *z* and log *P* were selected as the molecular descriptors and defined the extraction window of the liquid membranes. Both charge (derived from p*K*_a_ value) and log *P* are principal molecular descriptors, and values are found for a large number of substances in the literature. However, log *P* values determined experimentally may vary by one or more units and may be time-consuming to find. Alternatively, log *P* and charge can be obtained computationally, which is a great advantage since it is readily available and can be directly compared between analytes. Finally, charge and log *P* are common parameters that are familiar to scientists.

In the experimental work discussed below, a large number of model analytes were extracted with different liquid membranes, and for each experiment, recoveries were plotted as a function of analyte log *P*. The model analytes were mono- or dibasic small-molecule pharmaceuticals in the log *P* range of −4.2 to 8.1. To ensure that data were sufficient for generalization, 90 different model analytes were used. All experiments were conducted with sample pH 2.4, acceptor pH 2.4, an extraction potential of 50 V, and an extraction time of 30 min.

### Extraction with 2-Nitrophenyl Octyl Ether (NPOE), the Classical Liquid Membrane

**Figure 1:**
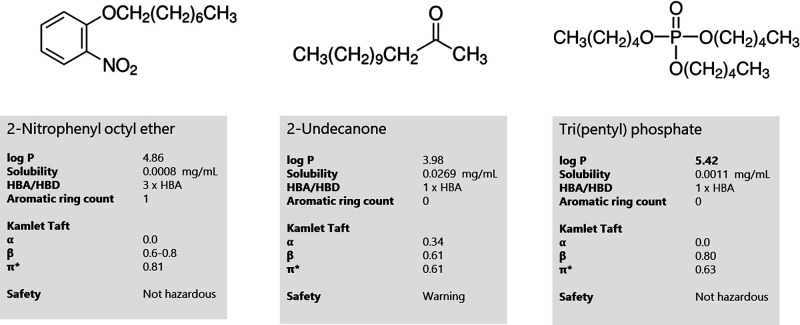
Chemical structures and physicochemical parameters of 2-nitrophenyl octyl ether, 2-undecanone, and tri(pentyl) phosphate.

In the first set of experiments, NPOE was tested as the liquid membrane. Its physiochemical properties are listed in Figure 1. NPOE has a log *P* of 4.86 and a solubility in water of 0.0008 mg/mL and is among the most hydrophobic liquid membranes used for EME.^17^ The aromatic ring count is one, and the number of hydrogen bond acceptor (HBA) sites is three (Figure 1). Kamlet–Taft parameters for NPOE could not be experimentally determined due to high background absorbance but have been reported in the literature as α = 0.0^19^ and π* = 0.81.^23^ A literature value for β was not found, but it is expected to be in the range of 0.6–0.8. NPOE has been hypothesized to interact with protonated basic analytes through hydrogen bond and cation−π interactions during EME.^14^

**Figure 2:**
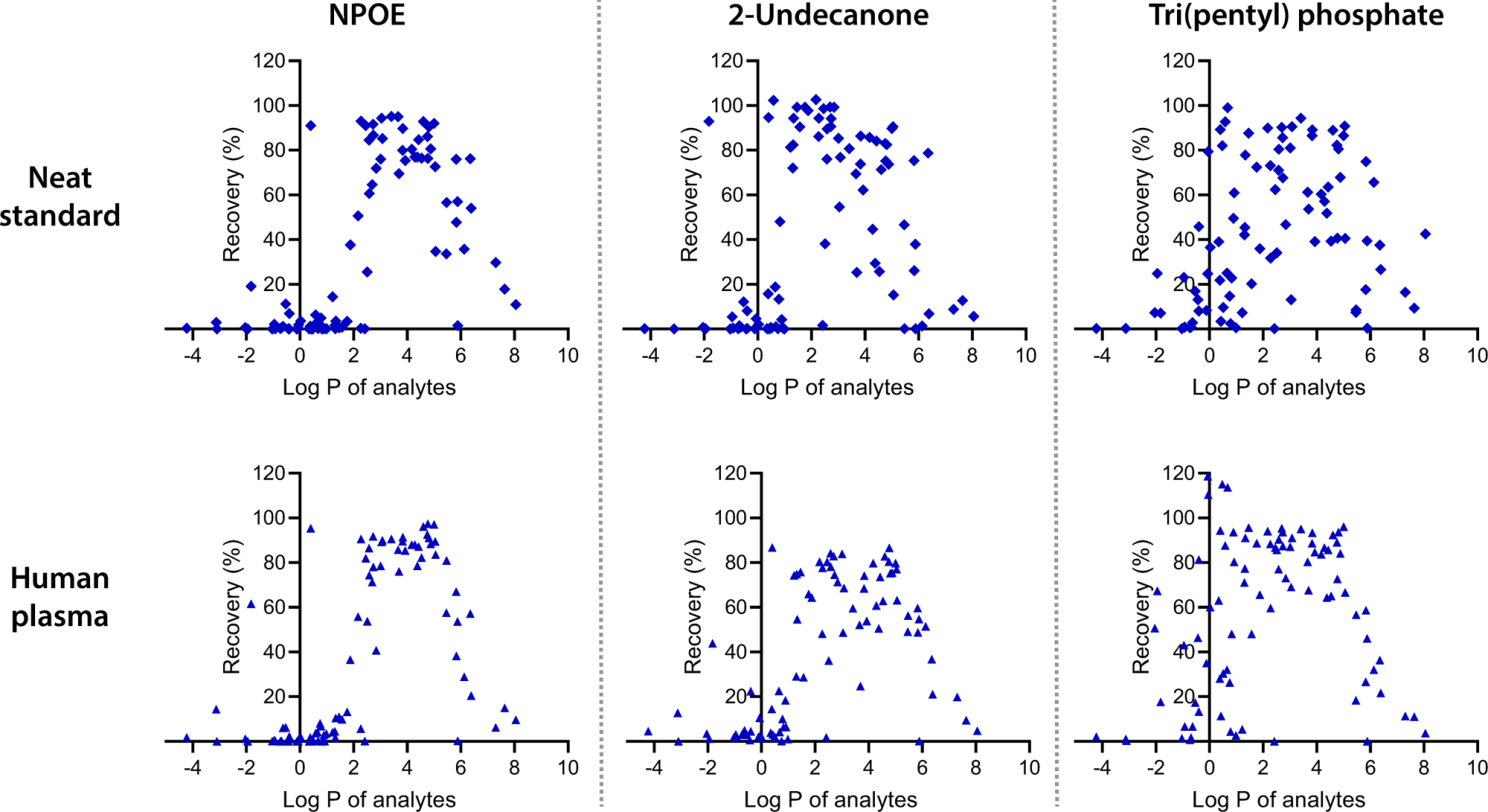
Extraction recoveries versus the log *P* value of 90 model bases from a neat standard (100 mM HCOOH) or spiked human plasma, using either NPOE, 2-undecanone, or tri(pentyl) phosphate as liquid membrane. Each point represents the average recovery of triplicate extraction. All extractions were performed at 50 V for 30 min.

Extraction recovery data from a neat solution for NPOE are summarized in Figure 2, where each data point corresponds to the recovery of an individual model analyte. From these data, the extraction window was defined according to the procedure described in “Definitions and Calculations” and is summarized in Table 1. In the range 2.2 ≤ log *P* ≤ 6.4, 84% of model analytes were extracted with recoveries above 40%. Model analytes with log *P* < 2.2 were in general too polar to enter the liquid membrane, and partition into the liquid membrane was not favored due to slow kinetics. Model analytes with log *P* > 6.4 were too lipophilic, and partitioning into the acceptor solution was not favored. As a result, the analytes suffered from membrane trapping. Inside the defined extraction window, analytes with *R* < 40% were explained by being close to the lower or upper limits of the window. Outside of the extraction window, one analyte, denatonium (log *P* = 0.41), was extracted with much higher efficiency (*R* = 91%) than other analytes of similar hydrophobicity. This was due to it being a quatenary ammonium compound (permanent cation), which cannot be neutralized and therefore has an artifically low log *P* value (log *P* is defined for neutral compounds). During extraction at pH 2.4, all analytes within the window had +1 ≤ *z* ≤ +2, except bumetanide (log *P* = 2.4, *z* = +0.6). Bumetanide was thus not extracted due to limited charge. No clear difference between mono- and dibasic analytes was observed. Exhaustive extraction was achieved for 30% of analytes within the window.

**Figure 3:**
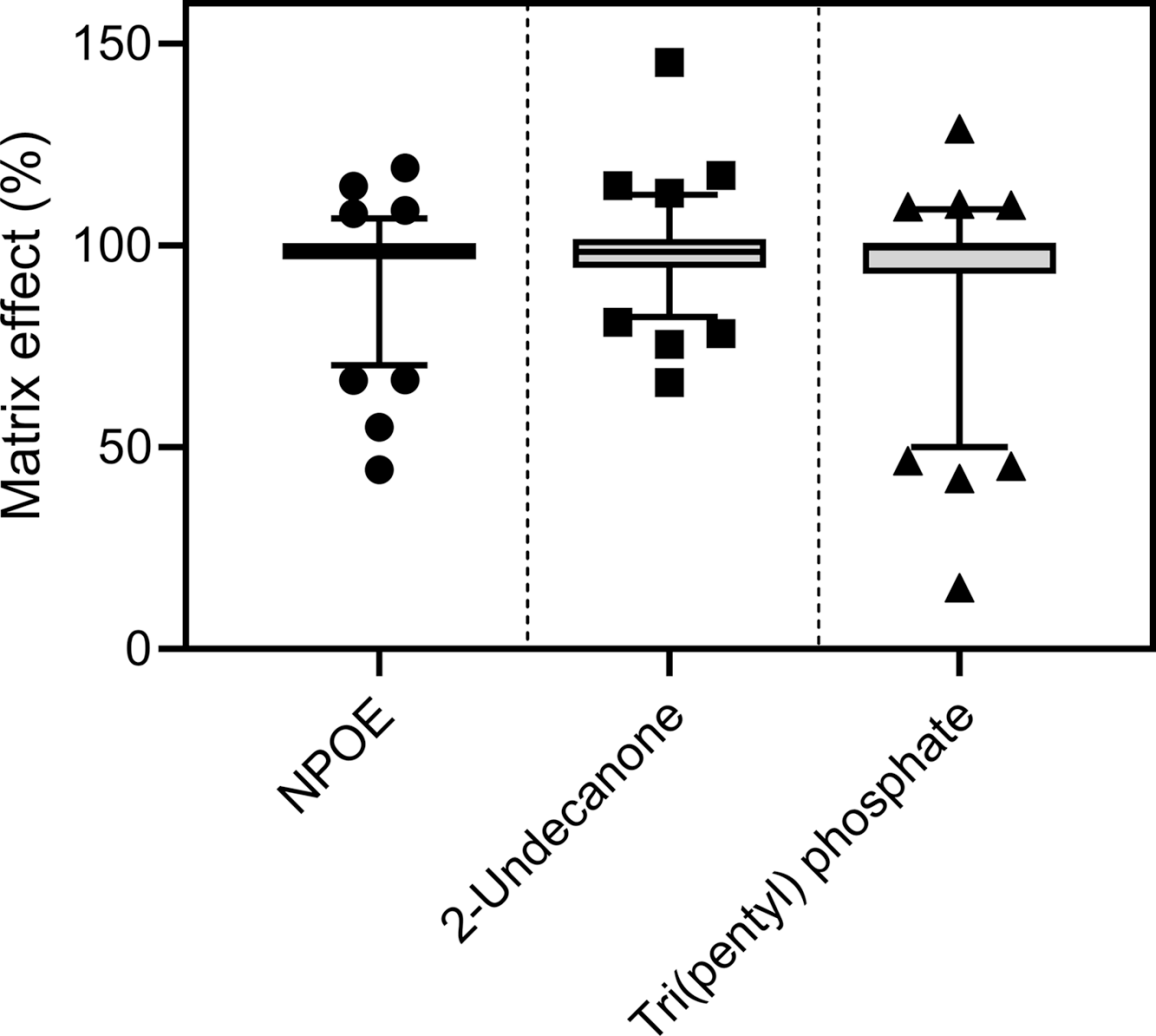
Box plots of matrix effects (%) for 90 model analytes in ESI-MS after EME from human plasma samples, using either NPOE, 2-undecanone, or tri(pentyl) phosphate as liquid membrane. Box plot whiskers represent 5 and 95% of observations, and individual points are outside this range. Matrix effects were calculated as the average of three replicates of postextraction spiked plasma. The relative standard deviation (RSD) for replicates was low, with an average RSD of 4.5–6.5% for the three liquid membranes.

**Table 1:** Extraction Window Data for NPOE, 2-Undecanone, and Tri(pentyl) Phosphate, Obtained from Neat Standard Solutions

SLM	NPOE	2-undecanone	tri(pentyl) phosphate
log *P*_Low_	2.2	1.0	0.5
log *P*_high_	6.4	5.8	5.5
average *R* within the window	68.4%	71.2%	57.7%
fraction >40% *R*	84%	83%	69%
fraction >85% *R* (exhaustive)	30%	41%	24%

**Figure 4:**
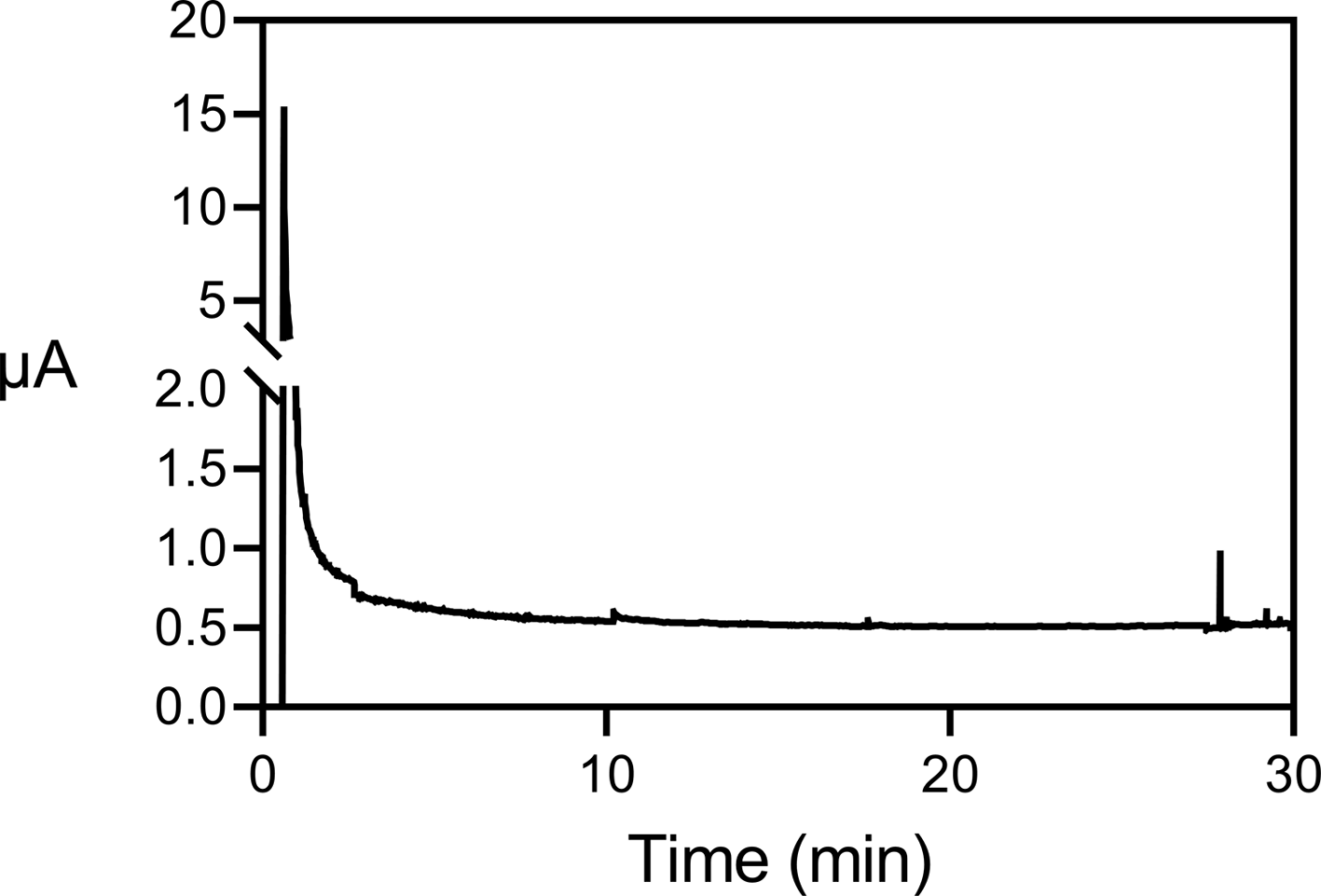
Extraction current (μA) during extraction of human plasma with NPOE as the liquid membrane. The current is recorded as the average current for six simultaneous extraction replicates.

Extractions from spiked human plasma gave very similar extraction performance to the neat standard sample, as seen in Figure 2, and the extraction window was unchanged. During EME from plasma, the extraction current was measured continously as function of time (current profile, Figure 4). Initially, the current was high due to the capacitive accumulation of charge but rapidly stabilized at 0.5–1 μA/sample when operated at 50 V.^24^ The current level was identical for spiked and unspiked plasma. At this low current, the number of protons involved in electrolysis in the sample and acceptor solution corresponded, by calculation, to about 0.1% of the protons available in the acceptor (100 mM HCOOH). Similarly, the volumes of produced H_2_ and O_2_ due to electrolysis corresponded, by calculation, to less than 0.1% of the total volumes of the sample and acceptor compartment. Thus, bubble formation and drifting pH were insignificant. Since the current was not increasing during extraction, the thickness of the liquid membrane remained constant, and water penetration into the liquid membrane was insignificant. NPOE thus fulfilled all criteria for generic liquid membranes in EME.

Based on NPOE water solubility, only 0.01% of the liquid membrane can dissolve in the acceptor solution during extraction. Despite this, by examination in the microscope, very small droplets of NPOE (emulsion) were occasionally observed on the surface of the acceptor after extraction. Therefore, the final amount of NPOE in the acceptor may be higher than what is expected from its water solubility and may impact the separation/detection by LC–MS/MS. In a final set of experiments, matrix effects (ME) in LC–MS/MS after EME of human plasma were therefore investigated according to Matuszewski et al.^20^ MEs for all 90 model analytes are summarized as box plots in Figure 3. As seen, the vast majority of analytes were very close to 100% (corresponding to no matrix effect). The average and median ME values were 96.0 and 99.0%, respectively, and 88% of analytes were within 80–120% ME as required by official bioanalytical method validation guidelines. The four analytes with the greatest ion supression were very hydrophobic and outside the extraction window of NPOE.

### Increasing Extraction Time and Voltage with NPOE

In a new set of experiments, the impact of increasing extraction time and voltage on the extraction window of NPOE was studied (Figure S2). When extraction time was increased from 30 to 60 min, extraction recoveries increased by about 10% for the model analytes inside the extraction window (2.2 ≤ log *P* ≤ 6.4). At the same time, extraction recoveries increased by about 50% for the model analytes of moderate polarity (log *P* < 2.2) outside the extraction window (data not shown). Due to slow kinetics of the latter, recoveries were still low after 60 min, and the extraction window essentially remained the same. When the voltage was increased from 50 to 100 V, extraction recoveries decreased about 10% for the majority of model analytes inside the extraction window, while an ∼100% increase was observed for the compounds outside the extraction window. Despite this, no additional model analytes were enhanced above the 40% recovery level. Based on the experiments, expansion of the extraction window for NPOE based on prolongation of extraction time, or based on higher extraction potential, was not successful.

### Screening for Alternative Membrane Solvents

Pure organic solvents were tested as alternative liquid membranes suitable for the model analytes of moderate polarity. Physicochemical properties (computational) of the solvents are summarized in Table S2. The solvents were aliphatic and aromatic ethers, alcohols, esters, phosphates, and nitro compounds. All solvents were nonvolatile and with low solubility in water (<0.5 mg/mL).

Extraction with these solvents was performed from spiked neat solution, and performance was evaluated based on their compliance with our acceptance criteria. Extractions using hexadecane and pentylbenzene were not successful due to criterion (1), indicating that liquid membranes solely capable of hydrophobic and π-type interactions are inefficient for EME. The remaining solvents all provided hydrogen bond basicity or acidity. Dihexyl ether and dodecyl acetate are both hydrogen bond acceptors (HBA), but their HBA properties are moderate, and their log *P* values are relatively high. Analytes could therefore not partition into the SLM, and these membrane solvents were eliminated according to criterion (1).

Of the tested alkylated phosphates, tri(pentyl) phosphate provided high recoveries, and current was acceptable. This solvent was in compliance with all three criteria and was selected for further experiments. Tris(2-ethylhexyl) phosphate (log *P* = 9.2) was also tested, but this membrane solvent was too hydrophobic and was rejected due to criterion (1). Further, tris(2-butoxyethyl) phosphate (log P = 3.9) was eliminated due to producing excessive current, and tri(butyl) phosphate (log *P* = 4.1) was avoided due to being potentially carcinogenic. Finally, 2-undecanone appeared as an interesting liquid membrane; this membrane solvent fulfilled all criteria and was selected for further investigation, as described below.

### Extraction with 2-Undecanone

2-Undecanone (Figure 1) has a log *P* of 4.0 and a water solubility of 0.03 mg/mL. The aromatic ring count is zero, and the number of hydrogen bond acceptor (HBA) sites is one. Kamlet–Taft parameters were measured as α = 0.34, β = 0.61, and π* = 0.61. Compared to NPOE, 2-undecanone is less dipolar and the hydrogen bond basicity (β) is lower. The data with 2-undecanone are summarized in Figure 2 and Table 1. With this membrane solvent, the extraction window was in the range 1.0 ≤ log *P* ≤ 5.8, with the greatest efficiency in the lower part of the range. 2-Undecanone was thus capable of extracting analytes of greater polarity than NPOE, as could be expected for a less hydrophobic solvent. Within the extraction window, 83% of analytes were extracted with recoveries above 40% from neat standard solution, and 41% were extracted exhaustively. Contrary to what was observed for NPOE, some dibasic analytes were extracted with less efficiency than monobasic ones. In example, dibasic substances quinine (log *P* = 2.5, *R* = 38%), pyrilamine (log *P* = 3.0, *R* = 55%), perphenazine (log *P* = 3.7, *R* = 25%), and prochlorperazine (log *P* = 4.4, R *=* 29%) had 2–3 fold lower recoveries than monobasic substances with similar hydrophobicity. For dibasic substances, it may therefore be recommended to use NPOE rather than 2-undecanone in the case of overlapping extraction windows.

Extraction from plasma gave a small decrease in efficiency for all analytes, as seen from Figure 2, and the extraction window was shifted slightly towards greater analyte hydrophobicity. The current with 2-undecanone (data not shown) was low and stable and ranged between 1–3 μA per sample during extraction at 50 V. LC–MS/MS matrix effects were similarly to NPOE found to be very limited, as seen from Figure 3, with an average and median ME of 98.1 and 98.5%, respectively. 96% of analytes were within 80–120% ME.

### Extraction with Tri(pentyl) Phosphate

Tri(pentyl) phosphate (Figure 1) has a log *P* of 5.4 and a water solubility of 0.001 mg/mL. The number of hydrogen bond acceptor (HBA) sites is one. Kamlet–Taft parameters were measured as α = 0.0, β = 0.80, and π* = 0.63. Compared to NPOE and 2-undecanone, tri(pentyl) phosphate is more hydrophobic and provides stronger hydrogen bond basicity (β). The extraction data with tri(pentyl) phosphate are summarized in Figure 2 and Table 1. With this liquid membrane, the extraction window was in the range 0.5 ≤ log *P* ≤ 5.5. However, as seen from Figure 2, the extraction selectivity was less correlated to analyte log *P* than for NPOE and 2-undecanone. Analytes (69%) within the window were extracted with recoveries greater than 40%, and 24% were exhaustive. Like for 2-undecanone, dibasic analytes exhibited worse extraction efficiency than corresponding monobasic ones. These were, in addition to the aforementioned analytes, procaine (log *P* = 1.9, *R* = 36%), hydralazine (log *P* = 0.8, *R* = 15%), and butylhydrazine (log *P* = 0.5, *R* = 10%). The latter two were, however, near the lower limit of the extraction window.

Interestingly, extractions from spiked plasma yielded overall better extraction performance than from neat standard solution, and analytes as polar as famotidine (log *P* = −2.0) and *N*-guanylurea (log *P* = −2.0) were extracted with greater than 40% recovery. Within the defined extraction window, the average recovery was 75%. In contrast to the two former membrane solvents, tri(pentyl) phosphate provided significantly higher current. When tri(pentyl) phosphate was operated at 50 V with plasma samples, the average current was 39 μA per sample (data not shown), which corresponded to a consumption of 3% of available protons in the acceptor solution. However, the current was relatively constant for 30 min, and this indicated that the liquid membrane was stable. Matrix effects were overall slightly greater with tri(pentyl) phosphate, as seen from Figure 3. The average and median ME values were 93.0 and 99.6%, respectively. Eighty-four percent of analytes were within 80–120% ME; however, the greatest ME was observed for the most polar and early eluting analytes that were outside the defined extraction window of tri(pentyl) phosphate. This further indicates that tri(pentyl) phosphate enables greater partitioning of more polar substances than NPOE and 2-undecanone.

### Repeatability and Concentration Dependence

**Figure 5:**
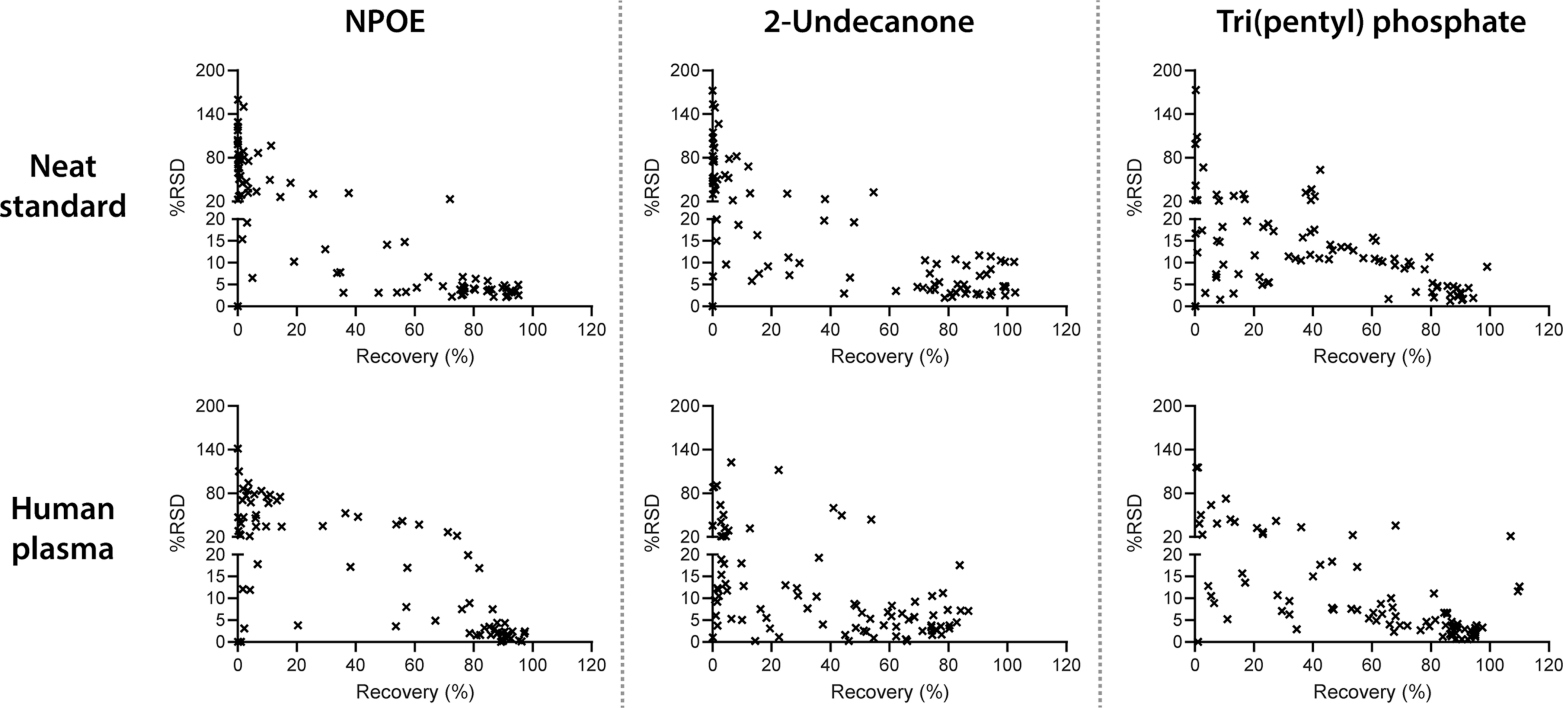
Relative standard deviation (%RSD) as a function of extraction recovery (Figure 2), based on triplicate extraction. Note that the *y*-axis is discontinuous for improved readability of low RSD values.

Repeatability of extraction data discussed above (Figure 2) is shown in Figure 5 as relative standard deviation (RSD) plotted against extraction recovery. As seen, repeatability improved considerably with increasing extraction recovery, and RSDs for analytes in the center of extraction windows (highest recoveries) were mostly less than 10%. No major differences in repeatability were observed between neat standards and plasma samples.

Next, the potential impact on extraction efficiency of different analyte concentrations was investigated by comparing recoveries obtained with NPOE (Table S3), 2-undecanone (Table S4), and tri(pentyl) phosphate (Table S5) from 1, 10, and 100 ng/mL samples. For each analyte, recoveries were compared by statistical testing (*t*-test or analysis of variance), and *p* values less than 0.05 were considered as significantly different recoveries. At the lower concentration levels, some analytes could not be quantified due to low recovery and/or poor response and were therefore omitted from statistical tests. For each liquid membrane, about 10 analytes were found to have a significant effect of concentration. However, there were no trends related to the concentration level or analyte hydrophobicity, and the absolute difference in recovery between concentrations was mostly small. The differences are therefore expected to arise from random variation.

### Molecular Interactions

During EME with NPOE, protonated bases are solvated in the liquid membrane due to a combination of strong hydrogen bond interactions and cation−π interactions. For this reason, NPOE is a very efficient and general liquid membrane for basic analytes. However, NPOE is hydrophobic (log *P* = 4.9), and due to this, NPOE is limited to bases of low polarity with log *P* ≥ 2.2. With 2-undecanone and tri(pentyl) phosphate, there are no cation−π interactions, and analyte solvation is mainly by hydrogen bond interactions. The absence of cation−π interactions may explain why 2-undecanone and tri(pentyl) phosphate exhibited decreased extraction performance for dibasic analytes, while there was no apparent effect with NPOE. For monobasic analytes, although the absence of cation−π interactions is unfavorable, this disadvantage is counteracted by the relatively low hydrophobicity of 2-undecanone (log *P* = 4.0). Due to this, even bases of moderate polarity can transfer into 2-undecanone, and this membrane solvent can therefore be used also for bases in the range 1.0 ≤ log *P* ≤ 2.0. Tri(pentyl) phosphate has a higher log *P* than NPOE (log *P* = 5.4), and solvation during extraction is facilitated by hydrogen bond interactions. Since tri(pentyl) phosphate provides high hydrogen bond basicity (β = 0.8) at multiple sites, monobasic analytes may overcome the greater hydrophobic barrier of the solvent. These hydrogen bond interactions were particularly important with decreasing analyte hydrophobicity. When calculating the ratio of HBD and HBA sites in each analyte, this ratio was increasing with decreasing log *P*, implying that polar analytes are more susceptible to be extracted by strong HBA liquid membranes. The lower range of tri(pentyl) phosphate’s extraction window could therefore be extended to log *P* = 0.5, and several analytes below this were also extracted. Further evidence to the importance of hydrogen bond interactions was seen for polar analytes with strong HBD sites. In example, phenolic groups are stronger HBD sites than aliphatic alcohols due to the delocalization of electrons in the aromatic ring. As such, famotidine (log *P* = −2.0, *R* = 25%), pyridoxine (log *P* = −1.0, *R* = 23%), sotalol (log *P* = −0.4, *R* = 46%), metaraminol (log *P* = 0.0, *R* = 79%), salbutamol (log *P* = 0.4, *R* = 39%), serotonin (log *P* = 0.5, *R* = 82%), and tyramine (log *P* = 0.7, *R* = 99%) all had strong HBD sites and were extracted with greater efficiency than other analytes of corresponding log *P*.

### Recommendations

From the discussions above, NPOE and 2-undecanone appear as appropriate candidates for generic liquid membranes for monobasic analytes with 1.0 ≤ log *P* ≤ 6.4. For overlapping extraction windows, NPOE is recommended as the first choice due to a large well-defined extraction window, high operational stability, and slightly better extraction performance from human plasma samples. NPOE can be expected to provide high extraction efficiency for most bases of low polarity within the log *P* range of 2.2 to 6.4. For bases of moderate polarity in the log *P* range of 1.0 to 2.0, NPOE is not very efficient and 2-undecanone is recommended. For bases with 0.5 ≤ log *P* ≤ 5.5, tri(pentyl) phosphate may be recommended as a third choice since the extraction window was less well-defined by analyte log *P* and z, and the operational current was higher. However, tri(pentyl) phosphate may have potential as a more selective liquid membrane and may even have potential in the range −1.0 < log *P* < 2.0. Selective liquid membranes may have important application areas and should be investigated more in the future. For dibasic analytes, NPOE is recommended as the first choice. It is emphasized that weak and partially charged bases may be difficult to extract despite being inside the defined extraction windows. In this case, a strongly acidic sample and acceptor solution may be used, as previously reported.^25^

The current recommendations and associated extraction performance are derived from prototype commercial EME equipment and are principally valid for this format that uses horizontal agitation. Conclusions regarding extraction selectivity (i.e., extraction windows) are, however, expected to be valid for other technical formats as well, such as 96-well^26^ or microfluidic devices,^27^ where geometries and means of convection are different. This is because the selectivity principally is governed by the hydrophobicity and chemical interactions between the SLM and analyte, which is independent of the equipment geometry and associated convection of aqueous solutions. Extraction efficiency within the extraction window, on the other hand, may vary between technical formats as this is strongly dependent on geometry and adequate convection.

## Conclusions

The current paper has initiated work to develop systems of generic extraction conditions for electromembrane extraction, based on analyte charge (*z*) and hydrophobicity (log *P*) as molecular descriptors. Attention was focused on bases of low and moderate polarity (log *P* > 0), and extraction recoveries were measured simultaneously for 90 different pharmaceuticals using prototype commercial EME equipment. This enabled generalization of data obtained with a number of different liquid membranes. From all candidates, 2-nitrophenyl octyl ether (NPOE), 2-undecanone, and tri(pentyl) phosphate are recommended as generic liquid membranes (in the given order). These solvents are nonvolatile, and their water solubility is low (≤0.03 mg/mL). NPOE was confirmed as the first choice for bases with log *P* in the range of 2.2–6.4 and provided high extraction efficiency and system stability. 2-Undecanone was discovered as a stable liquid membrane for bases in the log *P* range of 1.0–5.8. Both liquid membranes were stable for extraction from human plasma and provided low current. Tri(pentyl) phosphate is proposed as an alternative liquid membrane in the log *P* range of 0.5–5.5, with higher selectivity than what was observed for NPOE. Tri(pentyl) phosphate was also efficient for some model analytes in the log *P* range of −1.0 to 2.0.

In forthcoming research, the authors intend to describe similar data to those reported here for bases with log *P* < 1.0 and for acids. Although many papers have discussed EME of such substances, liquid membranes reported often lack the efficiency and stability requested in routine laboratories. The end goal is to provide a road map to EME, where robust generic extraction conditions can be found for compounds of interest based on charge and log *P*.

## Supporting Information Available

Photos of EME equipment; analytes, log *P*, charge, and LC–MS/MS parameters; list of tested liquid membrane solvents and their performance (PDF)
