# Supplementary material for: Generic Liquid Membranes for Electromembrane Extraction of Bases with Low or Moderate Hydrophilicity
Source: Anal Chem. 2023 Jun 1;95(23):8982–9. doi: 10.1021/acs.analchem.3c01052 (PMC10267886; doi:10.1021/acs.analchem.3c01052)
Supplement: Supplementary file 1 — ac3c01052_si_001.pdf [file ac3c01052_si_001.pdf]

Supporting information for

Generic liquid membranes for electromembrane extraction of bases with low or moderate hydrophilicity

Chen Zhou [a,b], Samira Dowlatshah [a], Anne Oldeide Hay [a], Maria Schüller [a], Stig Pedersen-Bjergaard [a,c], Frederik André Hansen [a] *

[a] *Department of Pharmacy, University of Oslo, P.O Box 1068 Blindern, 0316 Oslo, Norway*
[b] *West China School of Public Health and West China Fourth Hospital, Sichuan University, Chengdu, 610041, China*
[c] *Department of Pharmacy, Faculty of Health and Medical Sciences, University of Copenhagen, Universitetsparken 2, 2100 Copenhagen, Denmark*

* Corresponding author.

E-mail address f.a.hansen@farmasi.uio.no (Frederik André Hansen)

Content

Figure S1. Photos of EME prototype equipment.

Table S1. Model analytes and their log P, charge (z), and LC-MS parameters.

Table S2. Overview of tested membrane solvents and their performance.

Figure S1. Prototype equipment for EME. A) Conductive vials, support membrane union, and circular polypropylene membrane. B) Illustration of EME principle. BH^+, A^-, and N represent a protonated base, deprotonated acid, and a neutral substance, respectively. C) Assembled EME unit. D) 10-position vial holder and lid with electrode for each vial.

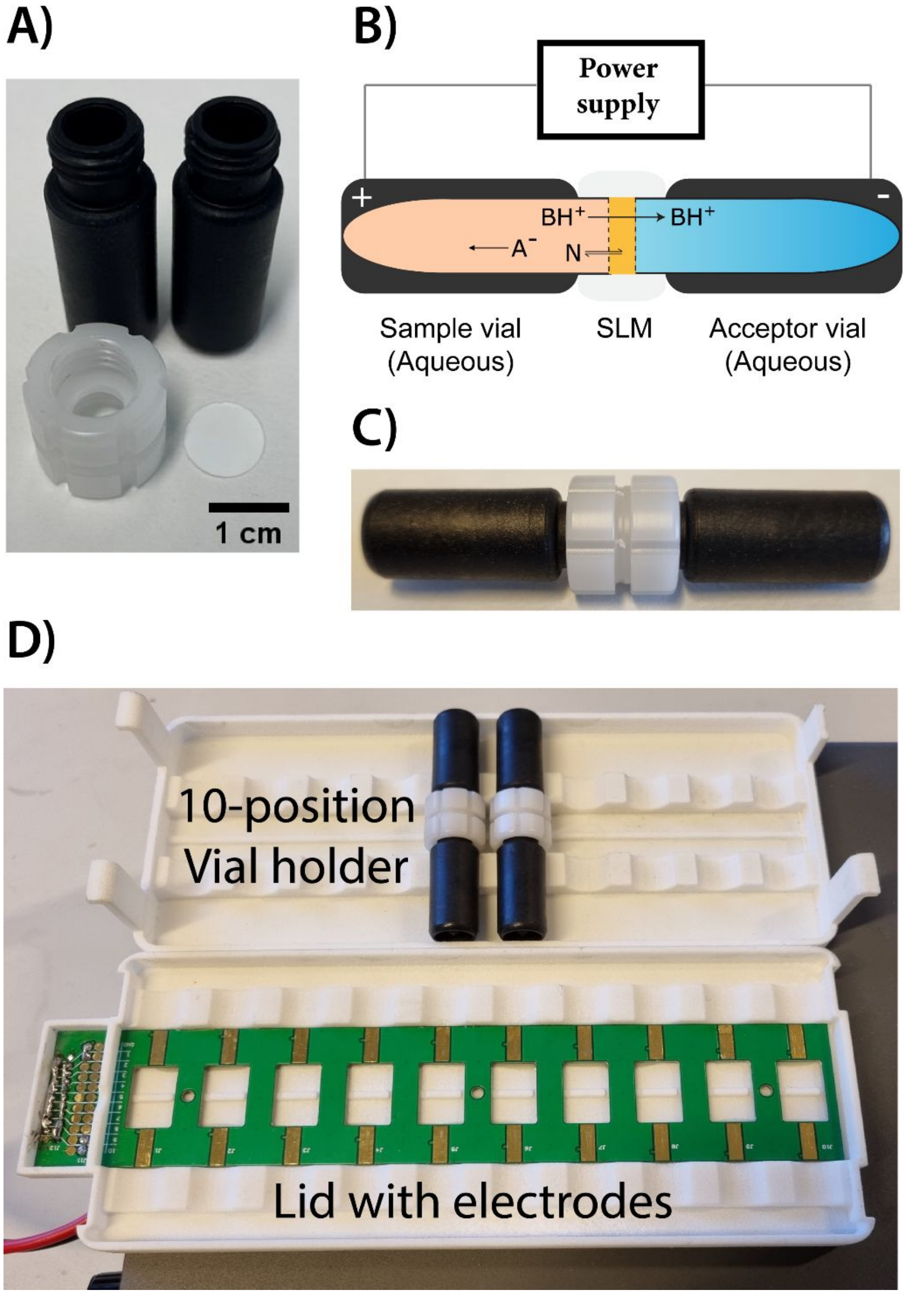

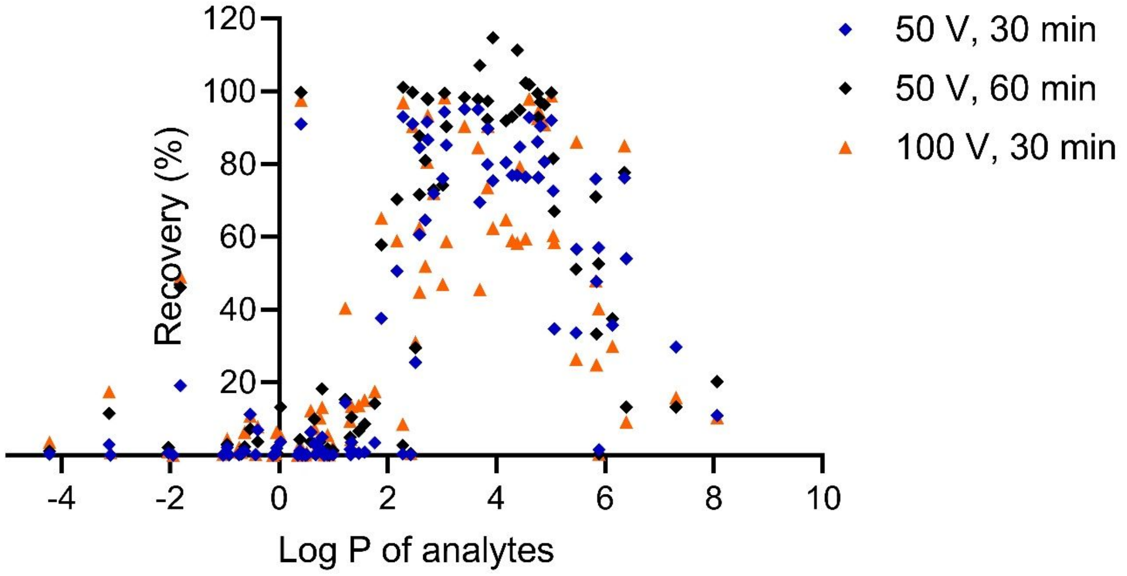

Figure S2. Effect of increasing extraction voltage and time from the initial parameters of 50 V for 30 minutes, with NPOE as liquid membrane.

Table S1. Model analytes and their log P, charge (z), and LC-MS parameters.

Compound	Producer	Log P	Z at pH 2.4	Retention time (min)	Precursor Ion (m/z)	Product Ion (m/z)	Collision energy (V)
**2,6-di-tert-butyl-4-(dimethylaminomethyl)-phenol**	Sigma–Aldrich (St. Louis, MO, USA)	4.6	1.00	4.62	264.2	219.2 (quantifier)	17
					264.2	203.2	33
**6-MAM**	Sigma–Aldrich (St. Louis, MO, USA)	1.31	1.00	2.43	328.2	211.1	29
					328.2	165.1 (quantifier)	49
**Acetyl choline**	Sigma–Aldrich (St. Louis, MO, USA)	-4.22	1.00	0.34	146	87 (quantifier)	13
					146	60.1	9
**Adenine**	Sigma–Aldrich (St. Louis, MO, USA)	-0.53	1.00	0.35	136.1	119 (quantifier)	25
					136.1	65	45
**Alprenolol**	Sigma–Aldrich (St. Louis, MO, USA)	2.69	1.00	3.93	250.41	173.1	17
					250.41	116.1 (quantifier)	17
**Amantidine**	Sigma–Aldrich (St. Louis, MO, USA)	1.47	1.00	2.55	152.1	135.1 (quantifier)	17
					152.1	77	50
**Amiodarone**	Sigma–Aldrich (St. Louis, MO, USA)	7.64	1.00	6.39	646	276.1	45
					646	201.1 (quantifier)	37
**Amitriptyline**	Sigma–Aldrich (St. Louis, MO, USA)	4.81	1.00	4.67	278.2	105	25
					278.2	91 (quantifier)	29
**Antipyrin**	Sigma–Aldrich (St. Louis, MO, USA)	1.22	0.0031	3.15	189.1	77 (quantifier)	49
					189.1	58.1	25
**Atenolol**	Sigma–Aldrich (St. Louis, MO, USA)	0.43	1.00	1.15	267.2	190.1	21
					267.2	145.1 (quantifier)	29
**Atropine**	Sigma–Aldrich (St. Louis, MO, USA)	1.57	1.00	2.78	290.41	124.1 (quantifier)	25
					290.41	93	33
**Benzamidine**	Sigma–Aldrich (St. Louis, MO, USA)	0.89	1.00	0.5	121.1	104 (quantifier)	21
					121.1	77	33
**Bumetanide**	Sigma–Aldrich (St. Louis, MO, USA)	2.42	0.66	6.04	365.1	240.1 (quantifier)	17
					365.1	184.1	25
**Butylhydrazine**	Sigma–Aldrich (St. Louis, MO, USA)	0.51	1.00	0.44	89.1	33.2 (quantifier)	9
					89.1	29.2	21
**Chlorpromazine**	Sigma–Aldrich (St. Louis, MO, USA)	4.54	1.00	4.86	319.1	246	25
					319.1	214.1 (quantifier)	49
**Chlorprothixene**	Sigma–Aldrich (St. Louis, MO, USA)	5.07	1.00	4.93	316.1	271 (quantifier)	21
					316.1	231	33
**Cimetidine**	Sigma–Aldrich (St. Louis, MO, USA)	-0.11	1.98	1.05	253.1	159.1	13
					253.1	95 (quantifier)	33
**Cinnarizine**	Sigma–Aldrich (St. Louis, MO, USA)	5.88	1.73	5.5	369.51	167.1 (quantifier)	21
					369.51	152.1	50
**Clofazimine**	Merck (Darmstadt, Germany)	7.30	1.41	6.2	473.1	431 (quantifier)	41
					473.1	395.1	50
**Clomipramine**	Sigma–Aldrich (St. Louis, MO, USA)	4.88	1.03	4.98	315.2	242.1	29
					315.2	227 (quantifier)	45
**Clotrimazole**	Sigma–Aldrich (St. Louis, MO, USA)	5.84	1.00	4.97	277	241.1	30
					277	165.1 (quantifier)	30
**Cocaine**	Norsk Medisinaldepot AS (NMD, Oslo, Norway)	2.28	1.00	3.33	304.2	182.1 (quantifier)	21
					304.2	105	37
**Denatonium**	Sigma–Aldrich (St. Louis, MO, USA)	0.41	1.00	4.27	326	234.2 (quantifier)	17
					326	112.1	29
**Diltiazem**	Sigma–Aldrich (St. Louis, MO, USA)	2.73	1.00	4.3	415.2	178 (quantifier)	25
					415.2	150.1	49
**Dopamine**	Sigma–Aldrich (St. Louis, MO, USA)	0.03	1.00	0.41	154.1	137.1 (quantifier)	9
					154.1	91	25
**Doxepin**	Sigma–Aldrich (St. Louis, MO, USA)	3.84	1.00	4.22	280.2	107 (quantifier)	25
					280.2	91	49
**Droperidol**	Sigma–Aldrich (St. Louis, MO, USA)	3.01	1.00	3.88	380.2	194.1 (quantifier)	13
					380.2	165.1	29
**Enalapril**	Sigma–Aldrich (St. Louis, MO, USA)	0.59	0.95	3.99	377.51	234.1 (quantifier)	17
					377.51	117	41
**Ephedrine**	Sigma–Aldrich (St. Louis, MO, USA)	1.32	1.00	1.35	166.1	148.1 (quantifier)	9
					166.1	91	37
**Epinephrine**	Sigma–Aldrich (St. Louis,	-0.43	1.00	0.34	184.1	166.1 (quantifier)	5

	MO, USA)				184.1	77	49
**Famotidine**	Sigma–Aldrich (St. Louis, MO, USA)	-1.95	1.02	0.98	338	259.1	9
					338	189 (quantifier)	21
**Fluoxetine**	Sigma–Aldrich (St. Louis, MO, USA)	4.17	1.00	4.83	310.1	148.1 (quantifier)	5
**Halofantrine**	Sigma–Aldrich (St. Louis, MO, USA)	8.06	1.00	6.53	500.4	142.1 (quantifier)	30
					500.4	100.1	30
**Haloperidol**	Sigma–Aldrich (St. Louis, MO, USA)	3.66	1.00	4.33	376.1	165.1 (quantifier)	25
					376.1	123	50
**Hydralazine**	Sigma–Aldrich (St. Louis, MO, USA)	0.75	1.04	0.6	161.21	89 (quantifier)	25
**Hydroxyzine**	Sigma–Aldrich (St. Louis, MO, USA)	3.41	1.82	4.61	375.2	201.1 (quantifier)	21
					375.2	166.1	50
**Ipratropium**	Sigma–Aldrich (St. Louis, MO, USA)	-1.82	1.00	2.93	332	166.2 (quantifier)	29
					332	124.1	37
**Isoniazid**	Sigma–Aldrich (St. Louis, MO, USA)	-0.69	1.09	0.35	138.1	121 (quantifier)	13
					138.1	79	33
**Lidocaine**	Sigma–Aldrich (St. Louis, MO, USA)	2.84	1.00	2.68	235.2	86.1 (quantifier)	17
**Loperamide**	Sigma–Aldrich (St. Louis, MO, USA)	4.77	1.00	5.24	478.11	267.2 (quantifier)	25
					478.11	210.1	50
**Luminol**	Sigma–Aldrich (St. Louis, MO, USA)	-0.06	0.14	2.33	178.1	105 (quantifier)	33
					178.1	78	45
**Meclizine**	Sigma–Aldrich (St. Louis, MO, USA)	6.39	1.84	5.75	391.2	201.1 (quantifier)	17
					391.2	166.1	50
**Mepiquat**	Sigma–Aldrich (St. Louis, MO, USA)	-3.12	1.00	0.35	114	98.1 (quantifier)	29
					114	58.1	29
**Metaraminol**	Sigma–Aldrich (St. Louis, MO, USA)	0.05	1.00	0.5	168.21	150.1 (quantifier)	5
**Metformin**	Sigma–Aldrich (St. Louis, MO, USA)	-0.92	2.00	0.33	130.1	71.1	25
					130.1	60.1 (quantifier)	13
**Methadone**	Norsk Medisinaldepot AS (NMD, Oslo, Norway)	5.01	1.00	4.71	310.2	265.2 (quantifier)	13
					310.2	105	33
**Metoprolol**	Sigma–Aldrich (St. Louis, MO, USA)	1.76	1.00	3.13	268.41	116.1 (quantifier)	17
					268.41	74.1	21
**Mianserin**	Sigma–Aldrich (St. Louis, MO, USA)	3.83	1.00	4.1	265.2	208.1 (quantifier)	21
					265.2	91	50
**N-acetylputrescine**	Sigma–Aldrich (St. Louis, MO, USA)	-1.03	1.00	0.33	131.1	114.1 (quantifier)	9
					131.1	72.1	13
**N-guanylurea**	Sigma–Aldrich (St. Louis, MO, USA)	-2.03	1.00	0.32	103.1	60.1 (quantifier)	9
					103.1	43.1	37
**Nicotinamide**	Sigma–Aldrich (St. Louis, MO, USA)	-0.39	0.95	0.44	123.1	80 (quantifier)	21
					123.1	53.1	33
**Nortriptyline**	Sigma–Aldrich (St. Louis, MO, USA)	4.43	1.00	4.6	264.41	105	21
					264.41	91 (quantifier)	25
**Noscapine**	Sigma–Aldrich (St. Louis, MO, USA)	2.58	1.00	3.65	414.2	353.1	25
					414.2	220.1 (quantifier)	21
**O-desmethylvenlafaxine**	Sigma–Aldrich (St. Louis, MO, USA)	2.27	1.00	2.87	264.2	246.2 (quantifier)	9
					264.2	107	41
**Oxprenolol**	European Pharmacopoeia Reference Standard	2.17	1.00	3.52	266.2	225.1	13
					266.2	116.1 (quantifier)	17
**Papaverine**	Sigma–Aldrich (St. Louis, MO, USA)	3.08	1.00	3.59	340.41	324.1 (quantifier)	33
					340.41	202.1	29
**Perphenazine**	Sigma–Aldrich (St. Louis, MO, USA)	3.69	1.92	4.61	404.2	171.1 (quantifier)	25
					404.2	143.1	29
**Pethidine**	Norsk Medisinaldepot AS (NMD, Oslo, Norway)	2.46	1.00	3.39	248.31	220.2 (quantifier)	21
					248.31	174.1	21
**Pimozide**	Sigma–Aldrich (St. Louis, MO, USA)	5.83	1.00	5.18	462.2	328.2 (quantifier)	33
					462.2	147.1	41
**Piperazine**	Sigma–Aldrich (St. Louis, MO, USA)	-0.73	2.00	0.28	87.1	44.1 (quantifier)	17
					87.1	27.2	45
**Practolol**	Sigma–Aldrich (St. Louis, MO, USA)	0.83	1.00	1.68	267.2	190.1 (quantifier)	17
					267.2	148.1	25
**Procaine**	Sigma–Aldrich (St. Louis, MO, USA)	1.88	1.60	1.51	237.31	100.1 (quantifier)	13
**Prochlorperazine**	Sigma–Aldrich (St. Louis,	4.38	1.95	4.72	374.1	141.2 (quantifier)	21

	MO, USA)				374.1	113.1	33
**Promazine**	Sigma–Aldrich (St. Louis, MO, USA)	3.93	1.00	4.42	285.1	180.1	49
					285.1	86.1 (quantifier)	17
**Promethazine**	Sigma–Aldrich (St. Louis, MO, USA)	4.29	1.00	4.34	285.41	198	25
					285.41	86.1 (quantifier)	13
**Propranolol**	Sigma–Aldrich (St. Louis, MO, USA)	2.58	1.00	3.88	260.2	183.1	17
					260.2	116.1 (quantifier)	17
**Pyridoxine**	Sigma–Aldrich (St. Louis, MO, USA)	-0.95	1.00	0.4	170.1	152.1 (quantifier)	9
					170.1	134.1	21
**Pyrilamine**	Sigma–Aldrich (St. Louis, MO, USA)	3.04	1.98	3.24	286.2	241.1	9
					286.2	121.1 (quantifier)	25
**Quinine**	Sigma–Aldrich (St. Louis, MO, USA)	2.51	2.00	2.72	163.1	189.1 (quantifier)	13
					163.1	117	41
**Raloxifene**	Sigma–Aldrich (St. Louis, MO, USA)	5.47	1.00	4.22	473.6	269	37
					473.6	112.1 (quantifier)	30
**Ranitidine**	Sigma–Aldrich (St. Louis, MO, USA)	0.99	1.00	1.23	315.1	176.1 (quantifier)	17
					315.1	130.1	25
**Reserpine**	Sigma–Aldrich (St. Louis, MO, USA)	3.53	1.00	4.97	609.3	397.2	29
					609.3	195.1 (quantifier)	41
**Salbutamol**	Sigma–Aldrich (St. Louis, MO, USA)	0.34	1.00	0.93	240.31	222.1	5
					240.31	148.1 (quantifier)	17
**Serotonin**	Sigma–Aldrich (St. Louis, MO, USA)	0.48	1.00	0.6	177.1	160.1 (quantifier)	5
					177.1	115	29
**Sotalol**	Sigma–Aldrich (St. Louis, MO, USA)	-0.4	1.00	1.01	273.01	255.1 (quantifier)	9
					273.01	133.1	29
**Sulfadiazine**	Sigma–Aldrich (St. Louis, MO, USA)	0.39	0.34	1.93	251.06	156	15
					251.06	92 (quantifier)	30
**Sulfamethazine**	Sigma–Aldrich (St. Louis, MO, USA)	0.65	0.34	2.99	279.1	124.1	25
					279.1	92 (quantifier)	33
**Sulfamethoxazole**	Sigma–Aldrich (St. Louis, MO, USA)	0.79	0.33	3.67	254.1	156	13
					254.1	92 (quantifier)	29
**Tamoxifen**	Sigma–Aldrich (St. Louis, MO, USA)	6.35	1.00	5.99	371.5	129.1	25
					371.5	72.27 (quantifier)	20
**Telmisartan**	Sigma–Aldrich (St. Louis, MO, USA)	6.13	1.94	4.82	258.1	305.1	9
					258.1	211 (quantifier)	9
**Thiamine**	Sigma–Aldrich (St. Louis, MO, USA)	-3.1	2.00	0.29	265	144.1	9
					265	122.1 (quantifier)	13
**Thioridazine**	Sigma–Aldrich (St. Louis, MO, USA)	5.47	1.00	5.2	371.2	258.1	29
					371.2	126.1 (quantifier)	25
**Timolol**	Sigma–Aldrich (St. Louis, MO, USA)	1.34	1.00	3.04	317.2	261.1 (quantifier)	13
					317.2	244.1	21
**Triclabendazole**	Sigma–Aldrich (St. Louis, MO, USA)	5.88	1.00	6.89	358.9	343.9	29
					358.9	273.9 (quantifier)	41
**Triisopropanolamine**	Sigma–Aldrich (St. Louis, MO, USA)	-0.63	1.00	0.38	192.2	174.2 (quantifier)	13
					192.2	98.1	21
**Trimipramine**	Sigma–Aldrich (St. Louis, MO, USA)	4.76	1.06	4.76	295.2	193.1	49
					295.2	100.1 (quantifier)	17
**Tyramine**	Sigma–Aldrich (St. Louis, MO, USA)	0.68	1.00	0.48	138.1	121.1 (quantifier)	5
					138.1	77	33
**Tyrosine methyl ester**	Sigma–Aldrich (St. Louis, MO, USA)	0.92	1.00	0.97	196.1	136.1	13
					196.1	91 (quantifier)	37
**Venlafaxine**	Sigma–Aldrich (St. Louis, MO, USA)	2.74	1.00	3.63	278.2	260.2 (quantifier)	9
					278.2	121.1	33
**Verapamil**	Sigma–Aldrich (St. Louis, MO, USA)	5.04	1.00	4.69	455.3	303.2	25
					455.3	165.1 (quantifier)	29

Table S2. Overview of tested membrane solvents and their performance.

Organic solvents	log P	Hydrogen bond acceptor count	Solubility (mg/mL)	Performance
Hexadecane	7.58	0	0.00	C
Pentyl benzene	4.27	0	0.02	C
2-Decanone	3.47	1	0.08	E
2-Undecanone	3.92	1	0.03	A
6-Undecanone	4.18	1	0.04	C
Tris(2-butoxyethyl) phosphate	3.95	4	0.05	B
Tri(butyl) phosphate	4.09	1	0.03	D
Tri(amyl) phosphate	5.42	1	0.00	A
Tris(2-ethylhexyl) phosphate	9.18	1	0.00	C
Bis(2-ethylhexyl) phosphite	5.78	3	0.00	A
Benzyl 2-nitrophenyl ether	3.48	3	0.02	B
2-Nitrophenyl pentyl ether	3.52	3	0.02	B
Dihexyl ether	4.55	1	0.02	C
2-Nitrophenyl octyl ether	4.86	3	0.00	A
2-Ethyl nitrobenzene	2.87	2	0.18	C
Iodopentafluorobenzene	3.62	0	0.04	C
2-nitro-cumene	3.16	2	0.07	C
Dodecyl acetate	4.80	1	0.00	C

* A = Recommended membrane solvent, B = Not recommended due to high/unstable current, C = Not recommended due to low extraction efficiency, D = Not recommended due to safety, E= Not recommended due to leakage,

Table S3. Recoveries at 1 ng/mL, 10 ng/mL, and 100 ng/mL using *NPOE* as liquid membrane. Compounds that that could not be quantified due to low recovery and/or response are marked <LOQ. p-values were calculated by one-way analysis-of-variance (ANOVA) when all three concentration levels were quantified, and t-test for when only the two upper levels were quantified. Compound only quantified at 100 ng/mL were not tested (-). Significantly different recoveries ($p < 0.05$) are marked with orange background.

Analyte	log P	1 ng/mL	10 ng/mL	100 ng/mL	p-value
2,6-di-tert-butyl-4-(dimethylaminomethyl)phenol	4.602	<LOQ	<LOQ	97.9	-
6-MAM	1.307	<LOQ	10.2	6.1	0.0407
Acetyl choline	-4.221	<LOQ	11.1	6.6	0.2649
Adenine	-0.531	<LOQ	<LOQ	2.2	-
Alprenolol	2.693	80.2	84.0	82.6	0.7479
Amantidine	1.466	<LOQ	19.4	11.9	0.0636
Amiodarone	7.635	<LOQ	<LOQ	53.0	-
Amitriptyline	4.81	96.2	83.0	94.3	0.1288
Antipyrin	1.219	14.2	23.6	16.8	0.0239
Atenolol	0.425	<LOQ	<LOQ	0.2	-
Atropine	1.571	16.0	20.4	13.2	0.3903
Benzamidine	0.894	18.3	5.9	4.0	0.1857
Bumetanide	2.423	<LOQ	10.2	12.4	0.1697
Butylhydrazine	0.514	<LOQ	<LOQ	0.2	-
Chlorpromazine	4.535	<LOQ	92.3	95.9	0.1817
Chlorprothixene	5.066	118.6	91.0	96.0	0.0924
Cimetidine	-0.109	<LOQ	<LOQ	0.1	-
Cinnarizine	5.88	78.5	66.7	79.7	0.2177
Clofazimine	7.304	<LOQ	57.7	42.3	0.9986
Clomipramine	4.883	97.8	85.1	91.4	0.4707
Clotrimazole	5.839	65.2	53.7	57.4	0.1188
Cocaine	2.282	90.2	89.9	96.7	0.1851
Denatonium	0.405	<LOQ	88.4	98.8	0.0563
Diltiazem	2.727	97.7	85.3	97.3	0.1756
Dopamine	0.03	<LOQ	<LOQ	3.8	-
Doxepin	3.84	96.7	85.7	93.5	0.1345
Droperidol	3.014	92.3	79.8	90.6	0.2694
Enalapril	0.588	<LOQ	21.1	12.3	0.0139
Ephedrine	1.318	<LOQ	5.2	3.1	0.1028
Epinephrine	-0.43	<LOQ	<LOQ	0.1	-
Famotidine	-1.95	<LOQ	<LOQ	0.2	-
Fluoxetine	4.173	<LOQ	84.9	93.9	0.0782
Halofantrine	8.057	<LOQ	<LOQ	70.2	-
Haloperidol	3.661	98.7	80.4	93.6	0.0199
Hydralazine	0.751	<LOQ	14.2	6.9	0.3962
Hydroxyzine	3.413	104.0	86.8	95.7	0.2296
Ipratropium	-1.818	79.1	83.2	78.5	0.9923
Isoniazid	-0.69	<LOQ	<LOQ	0.1	-
Lidocaine	2.843	63.4	73.8	60.1	0.3595
Loperamide	4.771	92.7	86.5	97.7	0.2621
Luminol	-0.064	<LOQ	<LOQ	0.3	-
Meclizine	6.388	<LOQ	59.6	48.2	0.8392
Mepiquat	-3.122	<LOQ	51.8	24.8	0.1207
Metaraminol	-0.045	<LOQ	<LOQ	3.1	-
Metformin	-0.918	<LOQ	<LOQ	1.5	-
Methadone	5.007	100.0	86.5	97.8	0.0846
Metoprolol	1.759	<LOQ	32.6	16.4	0.0714
Mianserin	3.831	100.1	88.4	92.4	0.0509
N-acetylputrescine	-1.026	<LOQ	<LOQ	0.5	-
N-guanylurea	-2.034	<LOQ	<LOQ	1.6	-
Nicotinamide	-0.394	<LOQ	<LOQ	9.4	-

Nortriptyline	4.426	94.6	79.2	92.2	0.0177
Noscapine	2.581	89.6	86.5	91.9	0.3750
O-desmethylvenlafaxine	2.274	11.8	15.5	10.4	0.4353
Oxprenolol	2.168	48.8	80.8	69.7	0.0366
Papaverine	3.08	93.5	82.0	93.7	0.0931
Perphenazine	3.692	<LOQ	89.7	104.2	0.0632
Pethidine	2.456	105.6	101.8	90.8	0.2895
Pimozide	5.826	<LOQ	94.1	93.3	0.3342
Piperazine	-0.729	<LOQ	<LOQ	0.5	-
Practolol	0.832	<LOQ	<LOQ	0.2	-
Procaine	1.88	49.4	64.1	46.4	0.2640
Prochlorperazine	4.382	<LOQ	91.7	103.9	0.0844
Promazine	3.931	112.9	101.8	95.7	0.0649
Promethazine	4.288	113.7	105.0	97.1	0.0604
Propranolol	2.584	79.9	77.5	88.4	0.0582
Pyridoxine	-0.951	<LOQ	<LOQ	0.8	-
Pyrilamine	3.044	108.6	88.2	94.6	0.0125
Quinine	2.513	<LOQ	54.9	31.5	0.0309
Raloxifene	5.465	<LOQ	77.3	92.3	0.0331
Ranitidine	0.991	<LOQ	<LOQ	0.2	-
Reserpine	3.531	<LOQ	<LOQ	95.1	-
Salbutamol	0.344	<LOQ	<LOQ	0.2	-
Serotonin	0.482	<LOQ	<LOQ	0.8	-
Sotalol	-0.395	<LOQ	31.3	2.7	0.2518
Sulfadiazine	0.387	<LOQ	3.1	2.2	0.5302
Sulfamethazine	0.65	<LOQ	<LOQ	5.1	-
Sulfamethoxalol	0.791	<LOQ	8.7	10.4	0.0234
Tamoxifen	6.351	<LOQ	<LOQ	64.2	-
Telmisartan	6.13	<LOQ	71.4	66.9	0.5988
Thiamine	-3.097	<LOQ	<LOQ	0.0	-
Thioridazine	5.469	59.0	74.0	83.3	0.2393
Timolol	1.336	14.4	19.1	14.1	0.2111
Triclabendazole	5.884	<LOQ	<LOQ	0.3	-
Triisopropanolamine	-0.63	<LOQ	26.2	7.1	-
Trimipramine	4.758	93.3	84.8	94.8	0.0577
Tyramine	0.68	<LOQ	4.4	0.6	0.2377
Tyrosine methyl ester	0.92	<LOQ	4.5	0.5	0.2498
Venlafaxine	2.739	85.9	87.0	92.3	0.1653
Verapamil	5.043	81.5	88.9	99.4	0.0203

Table S4. Recoveries at 1 ng/mL, 10 ng/mL, and 100 ng/mL using *2-undecanone* as liquid membrane. Compounds that that could not be quantified due to low recovery and/or response are marked <LOQ. p-values were calculated by one-way analysis-of-variance (ANOVA) when all three concentration levels were quantified, and t-test for when only the two upper levels were quantified. Compound only quantified at 100 ng/mL were not tested (-). Significantly different recoveries ($p < 0.05$) are marked with orange background.

Analyte	log P	1 ng/mL	10 ng/mL	100 ng/mL	p-value
2,6-di-tert-butyl-4-(dimethylaminomethyl)phenol	4.602	<LOQ	<LOQ	82.5	-
6-MAM	1.307	<LOQ	<LOQ	7.0	-
Acetyl choline	-4.221	<LOQ	<LOQ	1.8	-
Adenine	-0.531	<LOQ	<LOQ	5.4	-
Alprenolol	2.693	83.6	72.2	76.0	0.1589
Amantidine	1.466	26.1	22.7	36.7	0.3390
Amiodarone	7.635	<LOQ	<LOQ	9.4	-
Amitriptyline	4.81	102.8	81.8	80.9	0.0746
Antipyrin	1.219	18.0	10.8	9.2	0.0005
Atenolol	0.425	<LOQ	<LOQ	1.2	-
Atropine	1.571	5.6	3.0	5.4	0.3590
Benzamidine	0.894	19.4	8.0	14.6	0.0946
Bumetanide	2.423	<LOQ	<LOQ	12.3	-
Butylhydrazine	0.514	<LOQ	<LOQ	0.3	-
Chlorpromazine	4.535	<LOQ	<LOQ	89.8	-
Chlorprothixene	5.066	91.2	72.2	81.6	0.2876
Cimetidine	-0.109	<LOQ	<LOQ	0.4	-
Cinnarizine	5.88	73.8	60.9	71.2	0.3606
Clofazimine	7.304	<LOQ	<LOQ	59.9	-
Clomipramine	4.883	97.0	75.7	79.7	0.1160
Clotrimazole	5.839	88.8	66.8	67.1	0.0076
Cocaine	2.282	42.9	48.8	59.9	0.3257
Denatonium	0.405	86.1	80.4	83.8	0.4425
Diltiazem	2.727	75.4	68.4	77.6	0.2234
Dopamine	0.03	<LOQ	<LOQ	6.1	-
Doxepin	3.84	97.8	79.1	81.2	0.0607
Droperidol	3.014	72.3	68.3	69.9	0.7223
Enalapril	0.588	46.7	38.2	53.0	0.2829
Ephedrine	1.318	<LOQ	5.6	9.6	0.3805
Epinephrine	-0.43	<LOQ	<LOQ	0.3	-
Famotidine	-1.95	6.4	2.0	0.8	0.0813
Fluoxetine	4.173	89.8	81.8	79.1	0.2826
Halofantrine	8.057	<LOQ	<LOQ	42.5	-
Haloperidol	3.661	80.3	76.2	81.9	0.5082
Hydralazine	0.751	<LOQ	1.4	1.6	0.8364
Hydroxyzine	3.413	87.8	81.9	85.4	0.6275
Ipratropium	-1.818	9.1	6.6	9.6	0.6672
Isoniazid	-0.69	<LOQ	<LOQ	0.8	-
Lidocaine	2.843	18.5	16.8	29.7	0.2864
Loperamide	4.771	69.2	79.7	88.8	0.0386
Luminol	-0.064	<LOQ	<LOQ	17.0	-
Meclizine	6.388	218.8	69.7	47.5	0.1004
Mepiquat	-3.122	<LOQ	<LOQ	1.0	-
Metaraminol	-0.045	<LOQ	<LOQ	6.1	-
Metformin	-0.918	<LOQ	<LOQ	1.8	-
Methadone	5.007	97.8	81.7	83.4	0.0524
Metoprolol	1.759	56.7	26.1	30.1	0.1448
Mianserin	3.831	87.9	73.0	77.7	0.1131

N-acetylputrescine	-1.026	<LOQ	<LOQ	0.7	-
N-guanylurea	-2.034	<LOQ	<LOQ	2.7	-
Nicotinamide	-0.394	<LOQ	<LOQ	12.5	-
Nortriptyline	4.426	69.1	79.6	81.9	0.0672
Noscapine	2.581	67.6	62.2	76.2	0.2497
O-desmethylvenlafaxine	2.274	12.4	10.1	21.6	0.2977
Oxprenolol	2.168	68.1	56.5	64.2	0.5352
Papaverine	3.08	86.3	78.2	80.8	0.3627
Perphenazine	3.692	<LOQ	<LOQ	60.5	-
Pethidine	2.456	73.0	52.4	62.4	0.3673
Pimozide	5.826	<LOQ	<LOQ	70.6	-
Piperazine	-0.729	<LOQ	<LOQ	0.4	-
Practolol	0.832	<LOQ	<LOQ	1.0	-
Procaine	1.88	10.3	10.8	16.8	0.3478
Prochlorperazine	4.382	<LOQ	<LOQ	53.9	-
Promazine	3.931	39.2	83.3	85.2	0.0015
Promethazine	4.288	98.3	83.3	87.0	0.1791
Propranolol	2.584	82.8	71.9	75.7	0.1329
Pyridoxine	-0.951	<LOQ	<LOQ	3.1	-
Pyrilamine	3.044	8.4	22.6	32.3	0.0458
Quinine	2.513	<LOQ	<LOQ	15.4	-
Raloxifene	5.465	<LOQ	<LOQ	61.3	-
Ranitidine	0.991	<LOQ	<LOQ	0.5	-
Reserpine	3.531	<LOQ	<LOQ	53.3	-
Salbutamol	0.344	5.9	1.5	1.2	0.7778
Serotonin	0.482	<LOQ	<LOQ	5.6	-
Sotalol	-0.395	<LOQ	10.5	10.3	0.9725
Sulfadiazine	0.387	31.5	21.0	16.8	0.0628
Sulfamethazine	0.65	86.2	22.9	23.0	0.0014
Sulfamethoxalol	0.791	46.1	34.6	31.0	0.0145
Tamoxifen	6.351	<LOQ	<LOQ	51.1	-
Telmisartan	6.13	116.7	76.8	68.7	0.0067
Thiamine	-3.097	<LOQ	<LOQ	0.1	-
Thioridazine	5.469	36.0	43.4	73.3	0.0209
Timolol	1.336	19.3	14.7	29.3	0.1671
Triclabendazole	5.884	<LOQ	<LOQ	2.5	-
Triisopropanolamine	-0.63	<LOQ	<LOQ	11.8	-
Trimipramine	4.758	94.1	81.0	81.0	0.1650
Tyramine	0.68	<LOQ	<LOQ	3.5	-
Tyrosine methyl ester	0.92	11.5	4.1	3.5	0.3304
Venlafaxine	2.739	42.5	43.9	60.1	0.2103
Verapamil	5.043	125.0	88.7	90.4	0.0012

Table S5. Recoveries at 1 ng/mL, 10 ng/mL, and 100 ng/mL using *tri(pentyl) phosphate* as liquid membrane. Compounds that that could not be quantified due to low recovery and/or response are marked <LOQ. p-values were calculated by one-way analysis-of-variance (ANOVA) when all three concentration levels were quantified, and t-test for when only the two upper levels were quantified. Compound only quantified at 100 ng/mL were not tested (-). Significantly different recoveries ($p < 0.05$) are marked with orange background.

Analyte	log P	1 ng/mL	10 ng/mL	100 ng/mL	p-value
2,6-di-tert-butyl-4-(dimethylaminomethyl)phenol	4.602	<LOQ	<LOQ	82.5	-
6-MAM	1.307	<LOQ	<LOQ	7.0	-
Acetyl choline	-4.221	<LOQ	<LOQ	1.8	-
Adenine	-0.531	<LOQ	<LOQ	5.4	-
Alprenolol	2.693	83.6	72.2	76.0	0.1589
Amantidine	1.466	26.1	22.7	36.7	0.3390
Amiodarone	7.635	<LOQ	<LOQ	9.4	-
Amitriptyline	4.81	102.8	81.8	80.9	0.0746
Antipyrin	1.219	18.0	10.8	9.2	0.0005
Atenolol	0.425	<LOQ	<LOQ	1.2	-
Atropine	1.571	5.6	3.0	5.4	0.3590
Benzamidine	0.894	19.4	8.0	14.6	0.0946
Bumetanide	2.423	<LOQ	<LOQ	12.3	-
Butylhydrazine	0.514	<LOQ	<LOQ	0.3	-
Chlorpromazine	4.535	<LOQ	<LOQ	89.8	-
Chlorprothixene	5.066	91.2	72.2	81.6	0.2876
Cimetidine	-0.109	<LOQ	<LOQ	0.4	-
Cinnarizine	5.88	73.8	60.9	71.2	0.3606
Clofazimine	7.304	<LOQ	<LOQ	59.9	-
Clomipramine	4.883	97.0	75.7	79.7	0.1160
Clotrimazole	5.839	88.8	66.8	67.1	0.0076
Cocaine	2.282	42.9	48.8	59.9	0.3257
Denatonium	0.405	86.1	80.4	83.8	0.4425
Diltiazem	2.727	75.4	68.4	77.6	0.2234
Dopamine	0.03	<LOQ	<LOQ	6.1	-
Doxepin	3.84	97.8	79.1	81.2	0.0607
Droperidol	3.014	72.3	68.3	69.9	0.7223
Enalapril	0.588	46.7	38.2	53.0	0.2829
Ephedrine	1.318	<LOQ	5.6	9.6	0.3805
Epinephrine	-0.43	<LOQ	<LOQ	0.3	-
Famotidine	-1.95	6.4	2.0	0.8	0.0813
Fluoxetine	4.173	89.8	81.8	79.1	0.2826
Halofantrine	8.057	<LOQ	<LOQ	42.5	-
Haloperidol	3.661	80.3	76.2	81.9	0.5082
Hydralazine	0.751	<LOQ	1.4	1.6	0.8364
Hydroxyzine	3.413	87.8	81.9	85.4	0.6275
Ipratropium	-1.818	9.1	6.6	9.6	0.6672
Isoniazid	-0.69	<LOQ	<LOQ	0.8	-
Lidocaine	2.843	18.5	16.8	29.7	0.2864
Loperamide	4.771	69.2	79.7	88.8	0.0386
Luminol	-0.064	<LOQ	<LOQ	17.0	-
Meclizine	6.388	218.8	69.7	47.5	0.1004
Mepiquat	-3.122	<LOQ	<LOQ	1.0	-
Metaraminol	-0.045	<LOQ	<LOQ	6.1	-
Metformin	-0.918	<LOQ	<LOQ	1.8	-
Methadone	5.007	97.8	81.7	83.4	0.0524
Metoprolol	1.759	56.7	26.1	30.1	0.1448
Mianserin	3.831	87.9	73.0	77.7	0.1131

N-acetylputrescine	-1.026	<LOQ	<LOQ	0.7	-
N-guanylurea	-2.034	<LOQ	<LOQ	2.7	-
Nicotinamide	-0.394	<LOQ	<LOQ	12.5	-
Nortriptyline	4.426	69.1	79.6	81.9	0.0672
Noscapine	2.581	67.6	62.2	76.2	0.2497
O-desmethylvenlafaxine	2.274	12.4	10.1	21.6	0.2977
Oxprenolol	2.168	68.1	56.5	64.2	0.5352
Papaverine	3.08	86.3	78.2	80.8	0.3627
Perphenazine	3.692	<LOQ	<LOQ	60.5	-
Pethidine	2.456	73.0	52.4	62.4	0.3673
Pimozide	5.826	<LOQ	<LOQ	70.6	-
Piperazine	-0.729	<LOQ	<LOQ	0.4	-
Practolol	0.832	<LOQ	<LOQ	1.0	-
Procaine	1.88	10.3	10.8	16.8	0.3478
Prochlorperazine	4.382	<LOQ	<LOQ	53.9	-
Promazine	3.931	39.2	83.3	85.2	0.0015
Promethazine	4.288	98.3	83.3	87.0	0.1791
Propranolol	2.584	82.8	71.9	75.7	0.1329
Pyridoxine	-0.951	<LOQ	<LOQ	3.1	-
Pyrilamine	3.044	8.4	22.6	32.3	0.0458
Quinine	2.513	<LOQ	<LOQ	15.4	-
Raloxifene	5.465	<LOQ	<LOQ	61.3	-
Ranitidine	0.991	<LOQ	<LOQ	0.5	-
Reserpine	3.531	<LOQ	<LOQ	53.3	-
Salbutamol	0.344	5.9	1.5	1.2	0.7778
Serotonin	0.482	<LOQ	<LOQ	5.6	-
Sotalol	-0.395	<LOQ	10.5	10.3	0.9725
Sulfadiazine	0.387	31.5	21.0	16.8	0.0628
Sulfamethazine	0.65	86.2	22.9	23.0	0.0014
Sulfamethoxalol	0.791	46.1	34.6	31.0	0.0145
Tamoxifen	6.351	<LOQ	<LOQ	51.1	-
Telmisartan	6.13	116.7	76.8	68.7	0.0067
Thiamine	-3.097	<LOQ	<LOQ	0.1	-
Thioridazine	5.469	36.0	43.4	73.3	0.0209
Timolol	1.336	19.3	14.7	29.3	0.1671
Triclabendazole	5.884	<LOQ	<LOQ	2.5	-
Triisopropanolamine	-0.63	<LOQ	<LOQ	11.8	-
Trimipramine	4.758	94.1	81.0	81.0	0.1650
Tyramine	0.68	<LOQ	<LOQ	3.5	-
Tyrosine methyl ester	0.92	11.5	4.1	3.5	0.3304
Venlafaxine	2.739	42.5	43.9	60.1	0.2103
Verapamil	5.043	125.0	88.7	90.4	0.0012
